# Chemical Composition and Protective Effect of Young Barley (*Hordeum vulgare* L.) Dietary Supplements Extracts on UV-Treated Human Skin Fibroblasts in In Vitro Studies

**DOI:** 10.3390/antiox10091402

**Published:** 2021-08-31

**Authors:** Krystyna Joanna Gromkowska-Kępka, Renata Markiewicz-Żukowska, Patryk Nowakowski, Sylwia Katarzyna Naliwajko, Justyna Moskwa, Anna Puścion-Jakubik, Joanna Bielecka, Monika Grabia, Konrad Mielcarek, Jolanta Soroczyńska, Katarzyna Socha

**Affiliations:** Department of Bromatology, Faculty of Pharmacy with the Division of Laboratory Medicine, Medical University of Białystok, Mickiewicza 2D Str., 15-222 Białystok, Poland; renmar@poczta.onet.pl (R.M.-Ż.); patryk.nowakowski@umb.edu.pl (P.N.); sylwia.naliwajko@umb.edu.pl (S.K.N.); justyna.moskwa@umb.edu.pl (J.M.); anna.puscion-jakubik@umb.edu.pl (A.P.-J.); joanna.bielecka@umb.edu.pl (J.B.); monika.grabia@umb.edu.pl (M.G.); konrad.mielcarek@umb.edu.pl (K.M.); jolasor@interia.pl (J.S.); katarzyna.socha@umb.edu.pl (K.S.)

**Keywords:** young barley, antioxidant properties, UV radiation, skin care, nutricosmetics, fibroblasts, photoaging

## Abstract

Young barley seems to be a promising material for use as nutricosmetic due to the presence of many biologically active compounds. The aim of this study was to evaluate the effect of *Hordeum vulgare* L. extracts on human skin fibroblasts exposed to ultraviolet radiation B (UVB) radiation. Analysis of the chemical composition showed a predominance of 9,12,15-octadecatrienoic acid. The quality assessment showed that young barley preparations have high total polyphenolic content (TPC) and favourable total antioxidant status (TAS). They also contain antioxidant elements such as zinc, copper, and selenium. Furthermore, the analyzed products were found to be safe in terms of toxic elements (lead, cadmium and mercury) and lack of cytotoxic effect of young barley extracts on cells. In vitro bioactivity assays showed that young barley extract increased the survival rate and accelerated the migration of fibroblasts in research models with UVB radiation. The application of both extracts caused an increase in DNA biosynthesis, and in the number of cells arrested in S phase. Moreover, an inhibitory effect of the tested extracts on the expression of matrix metalloproteinase 2 (MMP-2) and matrix metalloproteinase 9 (MMP-9) was observed. The results indicate that young barley extracts, due to protective as well as restorative effect, could potentially be used in the production of nutricosmetics and skin care products.

## 1. Introduction

Skin, as the very sensitive and most external organ, is constantly exposed to damaging factors; it is the most vulnerable to anatomical and functional changes associated with aging. The process of skin aging is inseparably connected with changes in physical, morphological and physiological properties of individual skin layers. The essence of the aging process is changes in the histological image of the epidermis and dermis. The modifications stimulated by environmental (exogenous) conditions are superimposed on the changes occurring as a result of genetically determined modifications (chronological ageing) [1,2].

Prevention of long-term harmful effects of UV radiation on the skin can be minimized by using sunscreens, i.e., cosmetic products that contain UV filters as the crucial ingredient of the formula. Using sunscreens reduces the amount of UV to which the skin is exposed and reduces the occurrence of erythema and other changes leading to photoaging. Moreover, the use of such cosmetics prevents DNA damage in human skin cells, including melanocytes, and thus has a protective effect on senescent cells (SCC) and melanoma [3,4]. The mechanism of action of photoprotective products is based on the stimulation of the skin’s natural protection mechanisms, including melanogenesis and the formation of a barrier against UV radiation [5]. There is also the other side, the negative side of using sunscreens. Every year, 14,000 tons of sunscreens reach the waterways, and many of the chemicals involved in these cosmetic products, such as oxybenon and benzophenon-2, indicate an adverse effect on aquatic and terrestrial ecosystems, thus contributing to environmental change [6]. The solution to this problem seems to be to look for natural products that have a protective effect against UV radiation that show many positive effects on the skin, which could have less harmful effects on the environment.

The term “young barley” is applied to young leaf parts of the common barley plant—*Hordeum vulgare* L., belonging to the *Poaceae* family. It was found that young barley grass is characterized by a high content of nutrients, depending on the harvest time (growth phase), processing and storage [7]. Raw material is used for the production of dietary supplements that are appearing in two forms: barley grass powder or lyophilized juice. The young barley has significant amounts of non-enzymatic antioxidant factors such as vitamin C and E as well as β-carotene and chlorophyll. It contains minerals such as calcium, copper, iron, magnesium, potassium and zinc, and vitamins such as thiamine (B1), riboflavin (B2), pyridoxine (B6), pantothenic acid (B5), folic acid (B9) [8,9,10,11]. Young barley is promoted not only as a natural source of non-enzymatic but also enzymatic antioxidants, the most important of which are superoxide dismutase (SOD) and catalase (CAT), which have a protective function against the harmful effects of reactive oxygen species (ROS) [10]. There are many phenolic compounds found in barley, such as benzoic acid, cinnamic acid and its derivatives, proanthocyanids, quinones, flavonoids, chalcones, lavones, flavanones and aminophenolic compounds [12,13,14]. Young barley is a rich source of isoflavonoids, the most important of which are saponarin and 2-O-glycosyl isovitexin [15,16]. 

Limited scientific reports have indicated beneficial effects of various young barley formulations on skin. In a study conducted by Lee et al. [17], it was noted that oral supplementation of a fermented young barley and soybean formulation improved skin hydration while reducing stratum corneum thickness. In vitro tests demonstrated the ability of this formulation to inhibit hyaluronidase, which corresponded to increased levels of hyaluronic acid in both healthy fibroblasts and UV-treated keratinocytes [17]. In the study conducted in a hairless mouse model with a diet enriched with a mixture of fermented young barley and soybean, Kwon et al. [17] observed a significant decrease in UV-induced wrinkle formation by reducing collagen degradation and UVB-induced epidermal thickening due to decreased MMP-1 expression. Additionally, supplementation has been shown to improve skin barrier function and reduce oxidative stress by increasing SOD activity [18]. Meng et al. [17]] observed an inhibitory effect of methanolic barley grass extract on melanin biosynthesis in B16 melanoma cells. Tricin and its analogues (e.g., luteolin, tricetin and apigenin), present in young barley, were found to be compounds that act as an inhibitor of melanin secretion [19]. Another compound with studied inhibitory effects on melanogenesis is hordein which occurs naturally in barley, among others. An in vitro study using human epidermal melanocytes observed an inhibitory effect of hordein on melanin synthesis [20]. 

Protecting the skin from the sun’s radiation is extremely important. Nowadays, there is an increase in the impact of UVB radiation on the Earth, which is the result of the destruction of the ozone layer. This poses a serious danger to the skin as the risk of damage with long-term consequences such as photoaging, photoimmunosuppression and photocarcinogenesis [21]. The components of traditional sunscreens have a potential harmful effect on ecosystems and have the ability to accumulate in tissues, so it is necessary to look for other, more natural agents with both anti-aging and photoprotective effects. The aim of this study was to evaluate the protective effect of *Hordeum vulgare* L. extracts on human skin fibroblasts exposed to UV radiation in in vitro studies. Moreover, the quality assessment of young barley extracts was performed by determination of total polyphenolic content (TPC) and total antioxidant status (TAS), antioxidant (Zn, Cu, Se) and toxic (Pb, Cd, Hg) elements content and examination of chemical composition.

## 2. Materials and Methods

### 2.1. Materials

For the research, dietary supplements commercially available on the Polish pharmaceutical market were used: young barley juice powder (Greenie, Sopot, Poland), barley grass powder (NatVita, Mirków, Poland) and chlorophyll powder (Swanson Health Products, Fargo, ND, USA).

### 2.2. Determination of Mineral Components Content

The content of zinc (Zn) and copper (Cu) in the products from young barley (BG, BJ) and chlorophyll (Ch) was determined by atomic absorption spectrometry (ASA) with atomization in an acetylene-air flame. The content of selenium (Se), cadmium (Cd), and lead (Pb) with atomization in a graphite cuvette on a Z-2000 analyzer with Zeeman background correction. Mercury (Hg) content of the tested products was determined by AMA 254 atomic absorption spectrometer using amalgamation technique. Quality control was performed using certified reference materials—INCT-TL-1 (Institute of Nuclear Chemistry and Technology, Warsaw, Poland).

To the 0.25 g (±0.001 g) weights of young barley and chlorophyll prepared in teflon digestion dishes, 4 mL of concentrated spectrally pure nitric acid was added. The samples were mineralised by microwave technique in a closed system. Prepared minerals were transferred quantitatively to scintillation dishes using deionised water. Samples from Polish Certified Reference Material Tea Leaves (INCT-TL-1) were prepared similarly. Determinations were performed at the following wavelengths: λ = 213.9 nm (Zn), λ = 324.8 nm (Cu), λ = 196.0 nm (Se), λ = 283.3 nm (Pb), λ = 228.8 nm (Cd).

For the determination of Hg content, the samples did not require preliminary mineralisation. The content of Hg in preparations of young barley and chlorophyll was determined by the spectrophotometric method using the amalgamation technique directly in the studied material. Before analysis, nickel cuvette was prepared, thoroughly washed, rinsed three times in deionised water, dried and fired in a gas burner. Samples of young barley and chlorophyll of 0.02 g with an accuracy of 0.001 g were weighed into the cuvette and placed in the holder of the mercury analyser. After a drying and burning stage in a stream of oxygen, the mercury vapour separated from the sample was captured by an amalgamator. Then, the released mercury was determined by the ASA method at a wavelength of λ = 254 nm. The possibility of using the tested products as a source of the above elements in the diet was assessed on the basis of the obtained Zn, Cu and Se contents. For this purpose, the percentage of realization of the reference value intake (RVI) was calculated taking into account the maximum daily portions of the products recommended by the manufacturer in the leaflets, which were 5 g (BJ), 1 g (BG) and 0.5 g (Ch). According to current regulations [22], a product can be considered a source of a mineral when its daily serving covers 15% of the RVI for the element. For the toxic elements Cd, Pb and Hg, on the other hand, the results obtained were related to the legal maximum limits for the respective element: Cd—1000 µg/kg, Pb—3000 µg/kg, Hg—100 µg/kg [23,24,25].

### 2.3. Preparation of Extracts

Two types of extracts were prepared: 70% ethanolic (E) and water (W). 70% ethanol (*w*/*v*) and phosphate buffered saline pH 7.2(PBS) (Sigma, Burlington, MA, USA) were used for the preparation, respectively. Solvents were acidified with 0.1% trichloroacetate (TCA) (Sigma, Burlington, MA USA) before extraction. 

In order to prepare ethanolic extracts of young barley juice (EBJ), young barley grass (EBG) and chlorophyll (ECh), 10 g of each product, was weighed. The extraction was carried out on a rocker shaker (DOS-20 L Digital Orbital Shaker, ELMI, Newbury Park, CA, USA) at room temperature with no light with 100 mL of suitable solvent used in 50, 30 and 20 mL during the next three stages, lasting 3 h each. After each extraction cycle, the extract was centrifuged for 10 min (2000 rpm, room temperature) and filtered through a filter. The residue was poured again with a fresh portion of solvent. Portions of the extract were combined and evaporated on a rotary evaporator (Rotavapor R-3, Buchi, Essen, Germany) at 40 °C and then lyophilized (Alpha 1-2 LD plus, Christ, Osterode, Germany).

To make water extracts of young barley juice (WBJ), young barley grass (WBG) and chlorophyll (WCh) 3 g of the products were weighed, 30 mL of PBS was added, then the extract was intensely mixed, vortexed (Benchmark Scientific, Sayreville, NJ, USA) for 2 min, homogenized (3 × 30 s, 3000 rpm, room temperature) and frozen at −80 °C for 24 h. After that time, the extract was unfrozen at room temperature and homogenized (Omni International, Cambridgeshire, UK) again (3 × 30 s, 3000 rpm, room temperature). The extract was centrifuged (MPW Med.Instruments, Warsaw, Poland) (50 min, 5000× *g*, 4 °C), then the supernatant was collected and filtered through 0.45 µm and 0.2 µm syringe filters. In the finished extracts, the dry mass content was determined on a moisture analyzer (RadWag, Radom, Poland). The results are shown in a Table 1. Finished extracts were stored frozen at −20 °C until assayed.

### 2.4. Determinatio of Total Phenolic Content (TPC) 

The determination of total phenolic content (TPC) in the tested extracts was performed by the colorimetric method using Folin–Ciocalteu reagent, based on the spectrophotometric measurement at a wavelength of λ = 760 nm.

The tested ethanolic extracts (EBJ, EBG, ECh) were weighed at 0.01 g (±0.0001 g), dissolved in 70% ethanol in a volume of 10 mL and centrifuged for 5 min at 2000 rpm. From the water extracts (WBJ, WBG, WCh), 0.1 mL was taken and dissolved in 1 mL PBS and centrifuged for 5 min at 2000 rpm. From the centrifuged samples, 0.25 mL of the filtrate was taken, 1.25 mL of 0.2 N Folin–Ciocalteu reagent was added and stirred for 5 min. Subsequently, 1 mL of sodium carbonate solution was added, mixed and incubated for 2 h at room temperature in a dark place. The total phenolic content was expressed as mg gallic acid equivalents (GAE)/g. It was calculated from the calibration curve of standard solutions of gallic acid (in the range of 0 to 200 μg/mL). All tests were performed in triplicate.

### 2.5. Evaluation of Total Antioxidant Status (TAS) 

The determination of total antioxidant status (TAS) is based on the incubation of ABTS in the presence of peroxidase and dihydrogen dioxide, which leads to the formation of the ABTS+ radical. This radical has a relatively stable blue-green colour, which is measured at a wavelength of λ = 600 nm. The antioxidant substances contained in the test samples suppress the formation of this colour in proportion to their concentration. The lyophilized ethanolic extracts (EBJ, EBG, ECh) were weighed 0.01 g (±0.0001 g), dissolved in 1 mL of ethanol. From the prepared solutions of ethanolic extracts and aqueous extracts (WBJ, WBG, WCh) supernatant was pipetted into Eppendorf tubes. Three types of sample were prepared: blank, standard and samples by adding 10 µL DDH_2_O, 10 µL standard (6-hydoxy-2,5,7,8-tetramethylchroman-2-carboxylic acid), 10 µL test sample respectively. To each type of sample 500 µL of chromogen were added. 

All solutions were mixed and incubated at 37 °C for 10 min. The initial absorbance value (A_1_) was measured at λ = 600 nm. Then, 100 µL of substrate was added to each sample, mixed, and the absorbance (A_2_) was measured again after exactly 3 min. The difference in absorbance values (∆A) for the blank/standard/test sample was calculated from the equation:

∆A=A_2_ − A_1_ (1)

In the next step the coefficient value was calculated: 

coefficient = (lot specific standard concentration)/((∆A blank − ∆A standard))

Where:

∆A blank—difference between the initial absorbance and the one measured after 3 min for the blank,
∆A standard—difference between initial absorbance and measured absorbance after 3 min for standard

The total antioxidant status expressed in mmol/L was then determined:

TAS [mmol/L] = coefficient × (∆A blank − ∆A sample)(2)

### 2.6. Cell Culture

Normal human skin fibroblast (NHSF; CRL-1474) cell line was obtained from the ATCC collection (Rockville, MD, USA). The cells were cultured, to obtain confluence, in Dulbecco’s Modified Eagle Medium (DMEM) (Gibco, Waltham, MA, USA) with addition of 10% Fetal Bovine Serum (FBS) (Gibco, Waltham, MA, USA) and 1% solution of Penicillin-Streptomycin (Sigma, Burlington, MA, USA) in an incubator (Binder, Tuttlingen, Germany) ensuring proper hydration, temperature 37 °C and concentration of 5% CO_2_.

### 2.7. Exposure to UVB Radiation

The effect of young barley extracts on NHSF cells exposed to UVB radiation was assessed. The cell exposure scheme was the same for all performed in vitro assays. Prior to radiation, the cell plates were washed with PBS and then exposed to UV radiation, in fresh PBS-filled wells, using Crosslinker CL-100 (UVP Analytik Jena, Upland, CA, USA) at a dose 25 mJ/cm^2^ [26]. Then, the PBS layer was removed from the plates and fresh medium or the medium with extracts was added. The research was conducted in three experimental models:

- without UV radiation, as “control w/o UV”
- “UV 24 h”, radiation used after 24 h incubation with extracts in order to assess the protective effect of extracts,
- “UV 0 h”, radiation used before 24 h incubation with extracts where the repair properties of the test substances were assessed.

The results were also compared to the control without the extracts as “control w/o extracts”. Moreover, the effect of young barley extracts was compared to chlorophyll protective activity against UV radiation, previously reported in the available literature [27]. 

### 2.8. Cytotoxicity Assay

The influence of young barley (EBG, EBJ, WBG, WBJ) and chlorophyll (ECh, WCh) extracts on cell viability was evaluated using the MTT assay. Cells were seeded into 96-well plates in a volume of 200 μL/well, at the final density 2 × 10^4^ cells/well. The plates were incubated for 24 h at 37 °C and 5% CO_2_ to obtain confluence. To achieve appropriate dilutions of tested extracts (5, 10, 25, 50 and 100 µg/mL), “STOCK” solutions with concentration of 1000 µg/mL were prepared. We dissolved 10 mg of freeze-dried ethanolic extracts in DMSO (Dimethyl Sulfoxide) (Sigma, Burlington, MA, USA) and then transferred to DMEM. Taking into account the dry matter content of the extracts (Table 1), adequate volume of water extracts was taken and added directly to DMEM with 0.1% DMSO. To maintain constant conditions during the experiment, both medium (control) and solutions were replaced after 24 h. After 48 h, the medium was removed and the cells were rinsed with 200 mL of PBS (Sigma, Burlington, MA, USA) and MTT (3-[4,5-dimethylthiazol-2-yl]-2,5-diphenyl tetrazolium bromide) (Sigma, Burlington, MA, USA) dissolved in PBS (5 mg/mL) was added to the wells. After 2 h of incubation, the MTT solution was removed. The formed formazan was dissolved by adding 180 µL DMSO to each cavity and 20 µL Sorensen buffer. Absorbance was read by Multimode Plate Reader Victor X3 (PerkinElmer, Singapore) at 570 nm and viability was shown as a percentage of the control (medium with 0.1% DMSO).

### 2.9. DNA Biosynthesis

The influence of young barley extracts on DNA biosynthesis in NHSF cell line was carried out by radioisotope method by measuring the incorporation of tritium-labelled thymidine into cells’ DNA. Based on the results of the MTT test, only extracts from young barley products at concentrations of 25, 50 and 100 μg/mL were selected for thymidine incorporation. NHSF cells was seeded at a density of 1.5 × 10^5^ cells/well on 24-well plates. After obtaining 80–85% of confluence, the cells were treated with young barley extracts dissolved in DMEM with inactivated FBS and incubated for 44 h. After this time, 10 µL [^3^H]-thymidine (MP Biomedicals, Irvine, CA, USA) was added to each well and incubated again for 4 h. Next, medium was removed and the cell surface was washed twice with 1 mL of 0.05M cold tris(hydroxymethyl)aminomethane hydrochloride (Tris-HCl) (Sigma, Burlington, MA, USA) and 1 mL of 5% TCA. A total of 1 mL of sodium dodecyl sulfate (SDS) (Sigma, Burlington, MA, USA) was added to each well, and cell lysate was transferred to vessels with scintillator fluid (Sigma, Burlington, MA, USA). The incorporation of [^3^H]-thymidine into fibroblast DNA was measured using scintillation counter TriCarb 2810 TR (PerkinElmer, Singapore). The results are presented as a percentage of control.

### 2.10. Cell Cycle Assay

Changes in the NHSF cell cycle of treated with tested extracts and exposed to UVB radiation were observed using NucleoCounter NC-3000 (ChemoMetec, Allerod, Denmark) system. NHSF cells were seeded into 6-well plates at a density of 1 × 10^6^ cells/well and after incubation for 24 h were treated with EBG and WBJ extracts at a concentration of 25, 50 and 100 μg/mL. After 48 h of incubation, the research was carried out according to the 2-step cell cycle assay protocol of the manufacturer (ChemoMetec, Allerod, Denmark). Determination of the number of cells present in each cell cycle phase (subG1, G0/G1, S or G2/M) was performed by measuring fluorescently stained cells at 365 nm which was analyzed using NucleoView NC-3000 software (ChemoMetec, Allerod, Denmark).

### 2.11. Expression of Metalloproteinases

The expression of MMP-2 and MMP-9 was measured by gelatine zymography in the culture medium of NHSF. The cells on a culture dish at a density of 2.5 × 10^6^ cells were incubated with young barley extracts at a concentration of 25, 50 and 100 μg/mL for 48 h. After that, medium was collected in tubes and centrifuged in Vivaspin^®^ concentrators (Sartorius Stedim Biotech GmbH, Göttingen, Germany) (4000 rpm, 4 °C) to a concentration of up to 200 µL. In the concentrated medium, the protein content was determined by Lowry method. In the next steps, gels were prepared and the experiment was performed according to the method already described [28]. After completed electrophoretic separation, the gels were incubated for 30 min in 2% Triton X-100 (Sigma, Burlington, MA, USA) at 37 °C and then were treated for 18 h by substrate buffer (50 mM pH 7.8 Tris–HCl buffer with 5 mM CaCl_2_) (POCH, Sowińskiego, Poland) at 37 °C. After incubation, the gels were stained with ethanolic solution of Coomassie brilliant blue (Sigma, Burlington, MA, USA). The gel was destained by a solution of various concentrations of acetic acid (POCH, Sowińskiego, Poland), ethanol (POCH, Sowińskiego, Poland) and distilled water according to the method described by Frankowski et al. [29]. The stained gels were analysed using Intas Imaging Systems (Intas, Göttingen, Germany) to determine gelatinase activity based on the size of the discolored areas. Protein molecular weights were determined from a protein standard (SeeBlue Plus 2, Thermo Fisher, Waltham, MA, USA), used concurrently, throughout the experiment.

### 2.12. Wound Healing Assay (Scratch Test)

The effect of young barley extracts on the migration of NHSF cells was evaluated using a scratch test (wound healing test) by observing the extent to which cells overgrow the space created by removing part of the cells from the surface of the culture plate. 

A cell suspension at a density of 3 × 10^5^/mL was seeded into 6-well plates and placed in an incubator. After growth for 24 h, a line was made using a sterile tip, removing some of the cells. The culture was rinsed with sterile PBS, and then medium with 100 µg/mL young barley test extracts or DMEM with 0.1% DMSO, without extracts, was added as a control. Immediately after the addition of the medium, observation was performed using an Olympus CKX 41 inverted microscope with a digital video path and photographs were taken. Images were quantitatively analyzed using ImageJ 1.52V software. These steps was repeated after 24 and 48 h.

### 2.13. GC-MS Analysis of Young Barley

Purification of the extract (remove sugar compounds) was carried out on a Bakerbond Speedisk Octadecyl C18XF column (J.T. Baker, Avantor, Radnor, PA, USA). The disk was conditioned with methanol, water and again methanol. The extracts were weighted (2 g) and dissolved in water (20 mL), and the solutions were filtered through a disk. The substances retained on the disks were rinsed with 25 mL of diethyl ether, and residual water present in this solution was removed using a magnesium sulfate. Obtained solutions of the study substances in diethyl ether were allowed to evaporate, then taken up into vials with 2 mL of methanol and evaporated. Five milligrams of 70% ethanolic extract from young barley grass (EBG) were diluted with 220 μL of pyridine and 80 μL of BSTFA with an addition of 1% trimethylchlorosilane. The reaction mixture was sealed and heated for 0.5 h at 60 °C to form trimethylsilyl (TMS) derivatives.

GC-MS analyses of EBG were performed using GC-MS under the conditions previously described [30]. Based on the retention times of hexane solutions of n-alkanes (C8-C40), separated under the same determination conditions, and the retention times of the separated EBG components, linear programmed retention indices (IT) of gas chromatography were calculated. 

Two analytical parameters were used to identify the separated components: mass spectra and calculated retention indices. Identification of components was performed using an automated GC-MS data processing system provided by NIST 14 (NIST/EPA/NIH Library of Electron Ionization Mass Spectra). The identification was assumed to be valid if the results of the computer search of the mass spectra library were confirmed by experimental RI values.

### 2.14. Statistical Analysis

All data were analysed using Statistica software 13.0 (TIBCO Software, Palo Alto, CA, USA). The results were shown as a mean percent value of control ± standard deviation (SD) and are calculated from at least three independent experiments performed in triplicate. The normality of the data distribution was assessed using the Kolmogorov–Smirnov, Lilliefors, and Shapiro–Wilk tests. Significant differences calculated by means of Student-t tests. *p* values < 0.05 were accepted as statistically significant.

## 3. Results

### 3.1. Content of Mineral Components

Prior to performing the determinations, a method quality control was performed and the results are shown in Table 2. All results obtained were maintained within the required range of the certified value.

The average content of Zn, Cu and Se in tested products were determined by atomic absorption spectrometry. The results of the analysis are presented in Table 3. 

The highest average Zn content was observed in the juice of young barley (289.24 ± 12.87 mg/kg), and it was found that the daily portion of this juice recommended by the manufacturer (5 g) covers 15% of the RVI of Zn for adults [22]. The lowest content of this element (232.59 ± 7.07 µg/kg) was determined in the preparation of chlorophyll.

The highest mean content of Cu was found in chlorophyll (7722.6 ± 6.36 mg/kg) and a daily portion of this product, recommended by the manufacturer, meets the recommended RVI in 386.1%. The lowest content of this element (6.76 ± 0.09 µg/kg) was characterized by the preparation of barley grass.

In the case of Se, the highest average content was recorded in the chlorophyll preparation (Ch; 496.45 ± 12.87 µg/kg) and the lowest (28.46 ± 3.72 µg/kg) in young barley grass. The manufacturers’ recommended daily servings of all formulations tested covered the RVI for Se by only less than 1%. 

The Cd, Pb and Hg contents of the tested products were determined by atomic absorption spectrometry. The results of the analysis are presented in Table 4. 

Among the tested samples, the highest mean Cd concentration of 52.28 ± 5.33 µg/kg was found in chlorophyll preparation and the lowest, 26.84 ± 2.84 µg/kg, in preparation of young barley grass. None of the tested preparations exceeded the maximum permissible cadmium content (1000 µg/kg) set by the European Union Commission Regulation [23].

In the case of Pb, the average content in the tested products ranged from 0.62 ± 0.08 µg/kg (barley grass) to 257.24 ± 18.30 µg/kg (chlorophyll). None of the analyzed products exceeded the maximum permissible lead content (3000 µg/kg) set by the European Union Commission Regulation [24]. 

The Hg content in the analyzed samples was within the standard (100 µg/kg) specified in the European Union Commission Regulation [25]. The chlorophyll preparation had the highest average content (37.67 ± 4.65 µg/kg) and the lowest in young barley grass (7.95 ± 1.05 µg/kg).

### 3.2. Total Phenolic Content (TPC)

**Figure 1:**
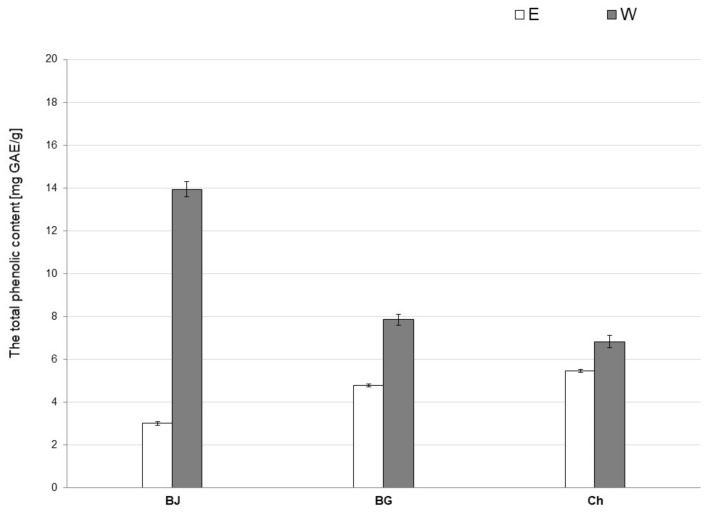
Total phenolic content (TPC) content of 70% ethanolic (E) and water (W) extracts from young barley juice (BJ), barley grass (BG) and chlorophyll (Ch).

The results of TPC determination of the tested extracts are shown in Figure 1. In the case of young barley juice extracts, the water extract (WBJ; 13.94 ± 0.35 mg GAE/g) had the highest mean polyphenol content. The TPC content in the 70% ethanol extract (EBJ) was 3.01 ± 0.08 mg GAE/g. Among the extracts prepared from barley grass, the mean TPC in water extract (WBG; 7.85 ± 0.24 mg GAE/g) was compared to 70% ethanolic extract (EBG; 4.79 ± 0.06 mg GAE/g). The mean TPC in extracts made from chlorophyll was higher in WCh (6.83 ± 0.28 mg GAE/g) than in ECh (5.45 ± 0.06 mg GAE/g). 

### 3.3. Evaluation of Total Antioxidant Status (TAS) 

**Figure 2:**
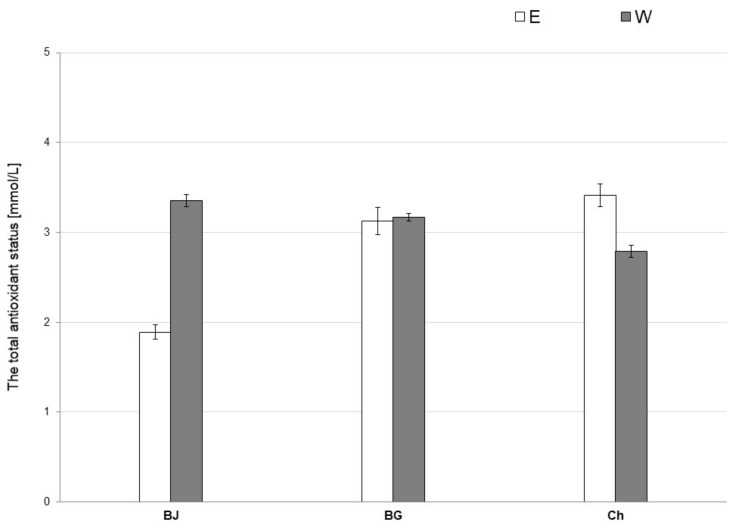
Total antioxidant status (TAS) of 70% ethanolic (E) and water (W) extracts from young barley juice (BJ), barley grass (BG) and chlorophyll (Ch).

The Kruskal–Wallis test showed a significant effect of the type of solvent used during extraction on the TAS value of the tested extracts. The results of TAS determination are shown in Figure 2. 

The water extract of young barley juice (WBJ) had a higher TAS value (3.353 ± 0.069 mmol/L) than the ethanol extract (EBJ; 1.888 ± 0.079 mmol/L). Moreover, for barley grass extracts, the TAS value was higher in WBG (3.170 ± 0.043 mmol/L) compared to EBG (3.124 ± 0.153 mmol/L). Among the chlorophyll extracts, the TAS value of ECh (3.414 ± 0.125 mmol/L) was higher than that WCh (2.789 ± 0.066 mmol/L).

### 3.4. Viability of Fibroblasts

**Figure 3:**
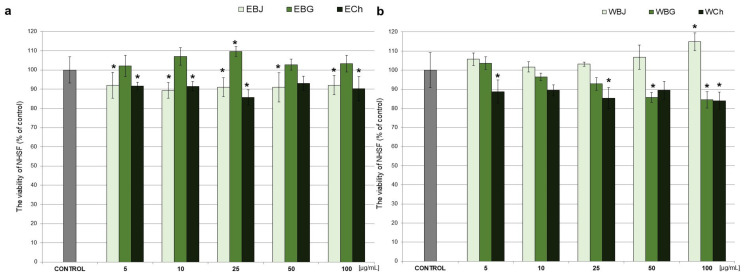
Effect of 70% ethanolic extract of young barley juice (EBJ), young barley grass (EBG) and chlorophyll (ECh) (**a**) and water extracts of young barley juice (WBJ), young barley grass (WBG) and chlorophyll (WCh) (**b**) at different concentrations (5, 10, 25, 50, 100 µg/mL) on the viability of normal human skin fibroblasts (NHSF) after 48 h incubation. The results are presented as percentage of control. * *p* < 0.05—statistically significant difference from control. Statistically significant difference between particular doses of the tested extracts (*p* < 0.05—EBG 5 vs. ECh 5, EBG 50 vs. ECh 50, EBJ 100 vs. EBG 100, WBG 10 vs. WCh 10, WBJ 50 vs. WCh 50, EBJ 10 vs. EBG 10, EBG 10 vs. ECh 10, EBJ 25 vs. EBG 25, WBJ 10 vs. WCh 10, WBJ 25 vs. WBG 25, WBJ 25 vs. WCh 25, WBJ 50 vs. WBG 50, EBG 25 vs. ECh 25, WBJ 100 vs. WBG 100, WBJ 100 vs. WCh 100).

Based on the in vitro MTT assay, the viability of human skin fibroblast cells treated with extracts from young barley (EBJ, EBG, WBJ, WBG) and chlorophyll (ECh, WCh) at different concentrations (5–100 μg/mL) was estimated and presented in Figure 3. 

Incubation of NHSF cell line with young barley juice extracts for 48 h (EBJ, WBJ) showed that EBJ significantly reduced fibroblast survival compared to controls in all doses (5–100 µg/mL), respectively, in the range 8–12%. EBG, compared to control cells, caused a significant increase in the survival of fibroblasts at a concentration of 25 µg/mL up to 10% (Figure 3a). An increase in cell survival (by 2–15% compared to controls) was observed after application of WBJ extract, and for a 100 µg/mL dose, it was statistically significant (*p* < 0.01). Relevant decrease in cell growth in comparison to the control cells caused WBG at concentrations of 50 and 100 µg/mL (Figure 3b). None of the chlorophyll extracts (ECh, WCh) caused a significant increase in cell survival. Application of ECh significantly decreased cell survival at doses of 5, 10, 25 and 100 µg/mL (up to 14%) (Figure 3a). WCh extract caused a significant decrease in cell survival compared to the control of 5, 25 and 100 µg/mL doses (in range 11–16%) (Figure 3b). The results also show the differences between particular doses of the tested extract. For ethanolic extracts, EBG at a dose of 25 µg/mL was most effective, and this was a statistically significant difference to both EBJ (*p* < 0.01) and ECh (*p* < 0.001). The strongest impact on the increase in NHSF viability, among water extracts, was the WBJ at 100 µg/mL (the statistically significant difference between WBJ vs. WBG and WCh was *p* < 0.001).

For further research, using UVB radiation on human skin fibroblasts, EBG and WBJ were selected. In order to check the potential protective and repair properties of extracts from young barley products, chlorophyll extracts in corresponding solvents (ECh, WCh) were used at the same time. 

**Figure 4:**
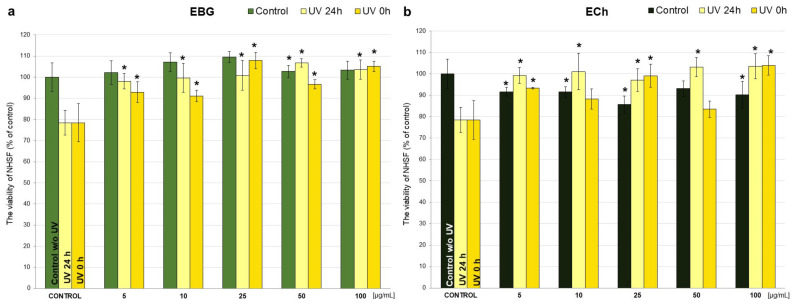
Effect of 70% ethanolic extract of young barley grass (EBG; (**a**)) and chlorophyll (ECh; (**b**)) at different concentrations (5, 10, 25, 50, 100 µg/mL) on viability of NHSF cells without and after application of UV radiation, after 48 h incubation. Results are presented as percentage of control. * *p* < 0.05—statistically significant difference from corresponding control. Statistically significant difference between particular doses of the tested extracts (*p* < 0.05 EBG 5 vs. ECh 5, EBG 50 vs. ECh 50, EBG 10 vs. ECh 10, EBG 50UV 0 h vs. ECh 50UV 0 h, EBG 25 vs. ECh 25).

**Figure 5:**
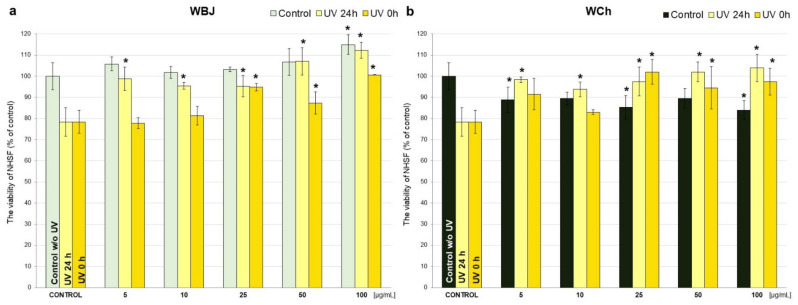
Effects of water extract of young barley juice (WBJ; (**a**)) and chlorophyll (WCh; (**b**)) at various concentrations (5, 10, 25, 50, 100 µg/mL) on the viability of NHSF cells without and after application of UV radiation, after 48 h incubation. Results are presented as percentage of control. * *p* < 0.05—statistically significant difference from corresponding control. Statistically significant difference between particular doses of the tested extracts (*p* < 0.05 WBJ 50 vs. WCh 50, WBJ 10 vs. WCh 10, WBJ 25 vs. WCh 25, WBJ 100 vs. WCh 100).

In order to study the effect of young barley extracts on NHSF treated with UVB radiation, the experiment was conducted in three experimental models as was described in Section 2.7 (Materials and Methods). UVB radiation applied to the NHSF resulted in a 22% decrease in the viability of these cells compared to control cells (which were not affected by any factor) (Figure 4 and Figure 5).

Ethanolic extracts caused a significant increase in cell viability (*p* < 0.001) compared to 24 h UV control without extracts in all doses, up to 28% for EBG at 50 µg/mL (Figure 4a) and 25% for ECh at 100 µg/mL (Figure 4b). No statistically significant differences were observed between the effects of barley grass extracts and chlorophyll. For the study of potential protective effect against UV radiation, EBG caused a significant increase in NHSF survival vs. UV 0 h control at all doses, with the best effect observed at 25 µg/mL (29%) (Figure 4a). The treatment with ECh in the “UV 0 h” model resulted in a significant increase in survival rate for doses of 5, 25 and 100 µg/mL (up to 25% for 100 µg/mL) (Figure 4b); the only significant difference between the extracts was observed for a dose of 50 µg/mL (EBG vs. ECh) (Figure 4). 

The use of water extracts before exposure of NHSF to UV radiation, both WBJ and WCh, resulted in a significant increase in cell survival, compared to 24 h UV control without extracts in all doses. The strongest effect was shown after applying 100 µg/mL dose, where the difference between the control was 34% for WBJ (Figure 5a) and 26% for WCh (Figure 5b). Similarly to ethanolic extracts, most doses of water extracts caused a significant increase in cell viability in the “UV 0 h” model. In case of WBJ, the doses of 25–100 µg/mL showed a significant increase in survival, whereas the highest effectiveness was shown by the dose of 100 µg/mL (increase by 22%). WCh significantly influenced the NHSF viability at doses of 5, 25, 50, 100 µg/mL. The most effective dose of 25 µg/mL causing 24% increase. No significant differences were observed between the effects of WBJ and WCh in both “UV 24 h” and “UV 0 h” (Figure 5).

The MTT test results allowed the selection of the most effective doses (25, 50, and 100 µg/mL) of extracts from young barley products (EBG and WBJ) for further studies.

### 3.5. The Influence of EBG and WBJ on DNA Synthesis 

**Figure 6:**
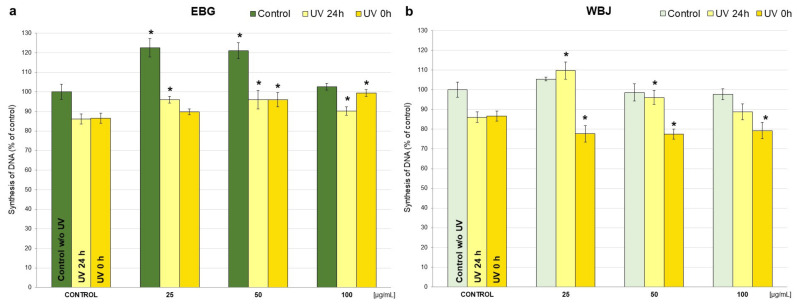
The effect of 70% ethanolic extract of young barley grass (EBG; (**a**)) and water extract from young barley juice (WBJ; (**b**)) at concentrations of 25, 50 and 100 µg/mL on the incorporation of [3H]-thymidine into DNA of healthy and UV-treated NHSF cells after 48 h incubation with tested substances. The results are presented as a percentage of the control. * *p* < 0.05—statistically significant difference from corresponding control. Statistically significant difference between individual doses of tested extracts (*p* < 0.05 EBG 25 vs. WBJ 25, EBG 50 vs. WBJ 50, EBG 25UV 24 h vs. WBJ 25UV 24 h, EBG 25UV 0 h vs. WBJ 25UV 0 h, EBG 50UV 0 h vs. WBJ 50UV 0 h, EBG 100UV 0 h vs. WBJ 100UV 0 h).

The assessment of cell proliferation was based on the level of [^3^H]-thymidine incorporated during DNA synthesis in fibroblast cells treated with EBG and WBJ extracts in doses 25, 50 and 100 µg/mL and UVB radiation. The research has shown that the application of UV radiation reduced the DNA synthesis in NHSF cell line by 14% compared to control without UVB on Figure 6. 

The treatment with EBG and WBJ on control without UV cells resulted in an increase in DNA synthesis for all doses used, whereas only for EBG 25 (23% increase; Figure 6a) and EBG 50 (21%; Figure 6b) was it statistically significant. In addition, the use of EBG at doses of 25 and 50 µg/mL resulted in significantly stronger stimulation of DNA synthesis than WBJ at the same doses (the difference is 17% and 22%, respectively). 

The determination of DNA biosynthesis in the “UV 24 h” test model evaluating the protective effects of the extracts showed significant changes after the application of EBG in all doses, an increase from 4% for 100 µg/mL to 10% for 25 and 50 µg/mL (Figure 6a). WBJ extract also caused a significant increase in DNA synthesis at doses of 25 µg/mL (by 24% compared to “UV 24 h” control) and 50 µg/mL (10%) (Figure 6b) The only significant difference between the effect of EBG and WBJ extract was observed for a dose of 25 µg/mL, and it was 14% of control.

A significant increase in DNA biosynthesis, in the “UV 0 h” model, was demonstrated after application of 50 µg/mL (*p* < 0.01) and 100 µg/mL doses of EBG (by 9% and 13%, respectively) (Figure 6a). The use of WBJ extracts resulted in a significant decrease in DNA synthesis in all doses and was 9% each for 25 µg/mL and 50 µg/mL and 7% each for 100 µg/mL (Figure 6b). The differences in the effect of EBG and WBJ extracts on DNA biosynthesis were statistically significant in the corresponding doses.

### 3.6. Cell Cycle Assay

**Figure 7:**
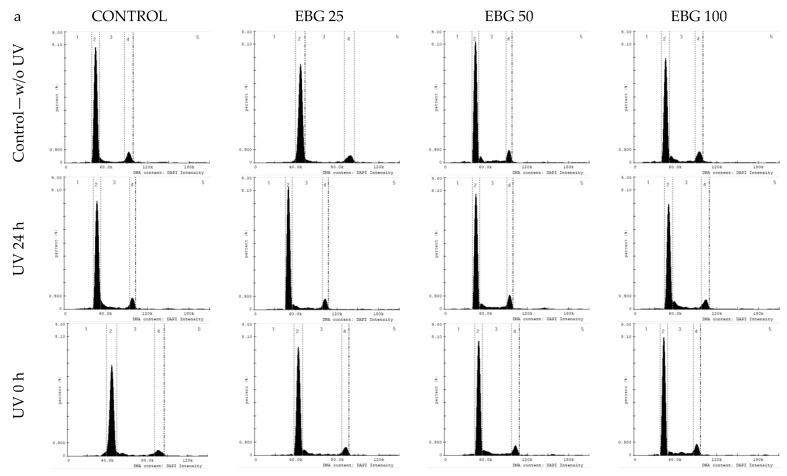

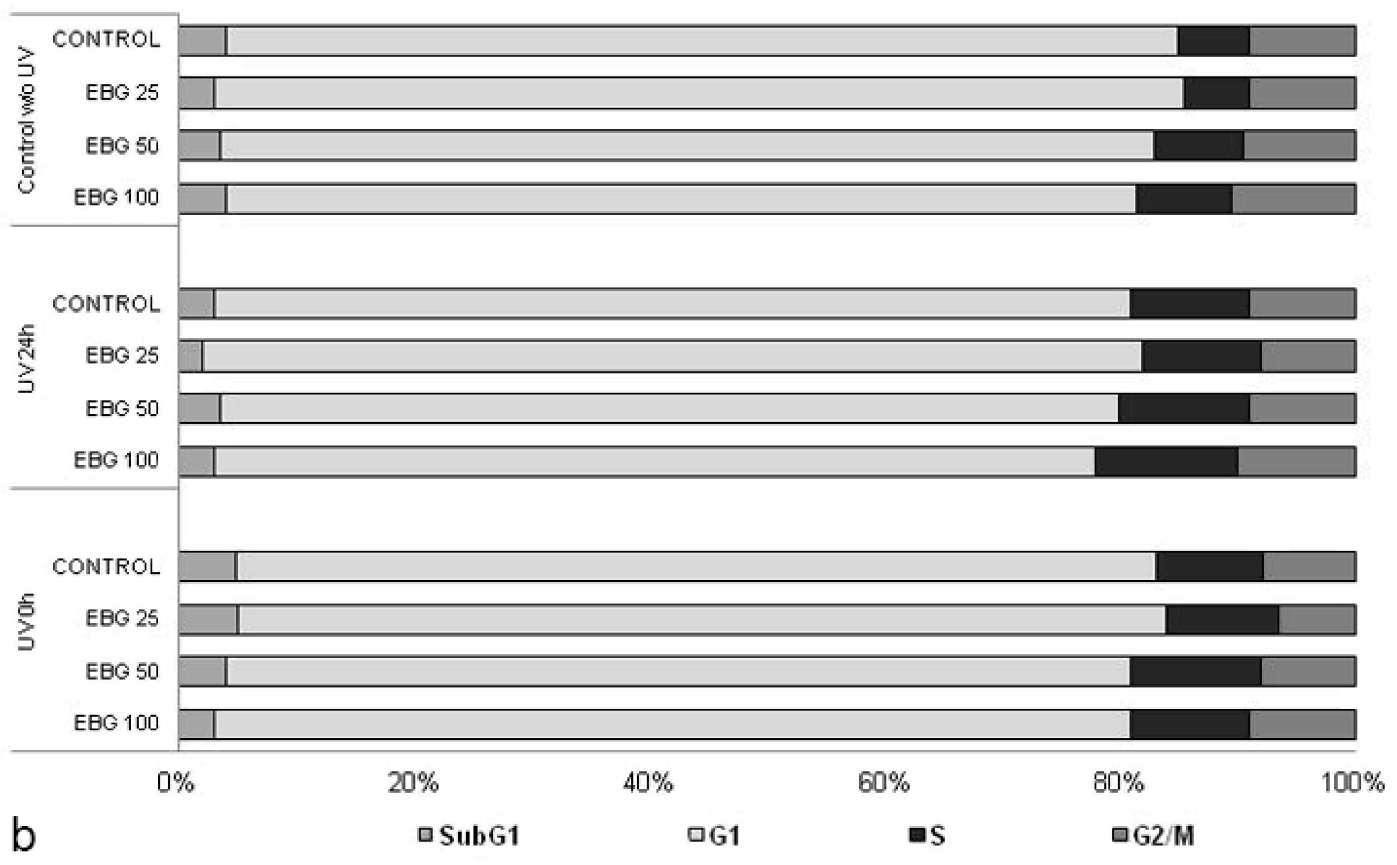
Analysis of cell cycle parameters in an NHSF cell line incubated for 48 h with 70% ethanolic barley grass extract (EBG) at concentrations of 25, 50 and 100 µg/mL. Histograms (**a**) illustrate the amount of DNA based on fluorescence intensity measurements. Graph (**b**) shows the percentage distribution of cells in each phase of the cycle compared to the control.

**Figure 8:**
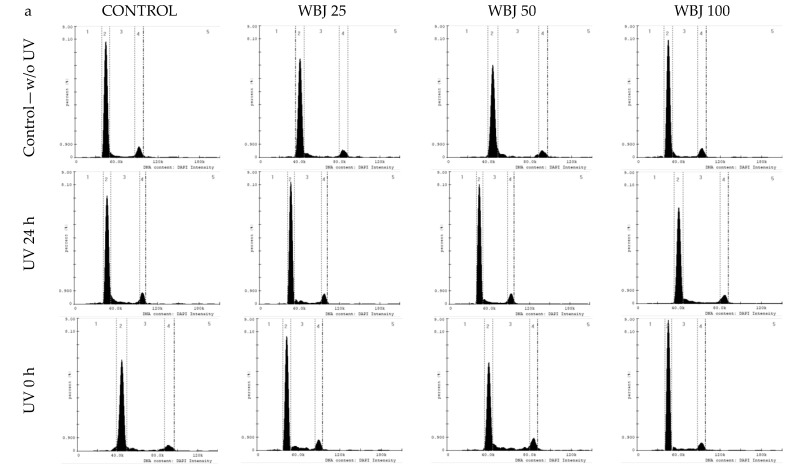

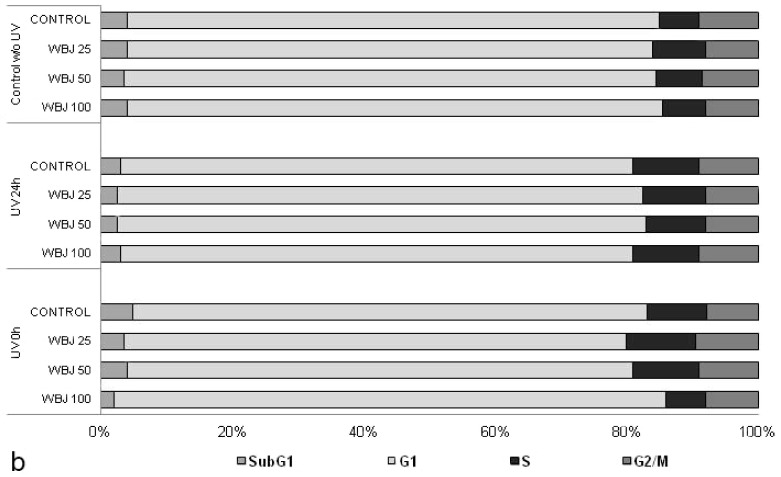
Analysis of cell cycle parameters in an NHSF cell line incubated for 48 h with water extract of young barley juice (WBJ) at concentrations of 25, 50 and 100 µg/mL. Histograms (**a**) illustrate the amount of DNA based on fluorescence intensity measurements. Graph (**b**) shows the percentage distribution of cells in each phase of the cycle compared to the control.

Treatment of control cells with UV light (without tested extracts) resulted in a decrease in the number of cells in G1 phase and an increase in S phase (Figure 7 and Figure 8).

Examining the effect of the extracts on cells without UV radiation, it was shown that EBG extract at doses of 25 and 50 µg/mL caused a decrease in the number of cells in the SubG1 phase compared to the control without extracts. Doses of 50 and 100 µg/mL decreased the number of cells in G1 phase and increased the number of cells arrested in S and G2/M phase (Figure 7). For the WBJ extract, a decrease in the number of cells in the SubG1 phase was observed after the 50 µg/mL dose. A dose of 25 µg/mL resulted in a decrease in the number of cells arrested in G1 phase. All doses used resulted in an increase in the number of cells in S phase and a decrease in G2/M phase (Figure 8).

In the “UV 24 h” test model, EBG extract at a dose of 25 µg/mL caused a reduction in the number of cells in the SubG1 and G2/M phases with an increase in the number of cells in the G1 phase compared to the control without extracts. After a dose of 50 µg/mL of this extract, an increase in the number of cells retained in S phases and a decrease in G1 phase were observed. A decrease in the number of cells in G1 phase also occurred after a dose of 100 µg/mL. At the same time, this dose resulted in an increase in the number of cells arrested in G2/M phase (Figure 7). WBJ extracts at doses of 25 and 50 µg/mL resulted in a decrease in the number of cells in S and G2/M phase while increasing the number of cells in G1 phase, compared to the UV 24 h control without extracts. The 100 µg/mL dose had no effect on fibroblast cell cycle (Figure 8).

EBG extract, in the “UV 0 h” model, at doses of 50 and 100 µg/mL caused a decrease in the number of cells arrested in SubG1 and G1 phases with an increase in the number of cells in S phase compared to the UV 0 h control. The 25 µg/mL dose caused a decrease in the number of cells in the G2/M phase, while the 100 µg/mL dose increased the number of cells in this phase (Figure 7). For the WBJ extract, all doses caused a decrease in the number of cells in the SubG1 phase. A decrease in the number of cells arrested in the G1 phase was observed with the 25 and 50 µg/mL doses, while the 100 µg/mL dose caused an increase over the 0 h UV control without extracts. The 25 and 50 µg/mL doses contributed to the increase in the number of cells arrested in S and G2/M phases. WBJ at a dose of 100 µg/mL caused a decrease in the number of cells in S phase while having no effect on the number of cells in G2/M phase (Figure 8).

### 3.7. The Influence of EBG and WBJ on MMP Expresion

**Figure 9:**
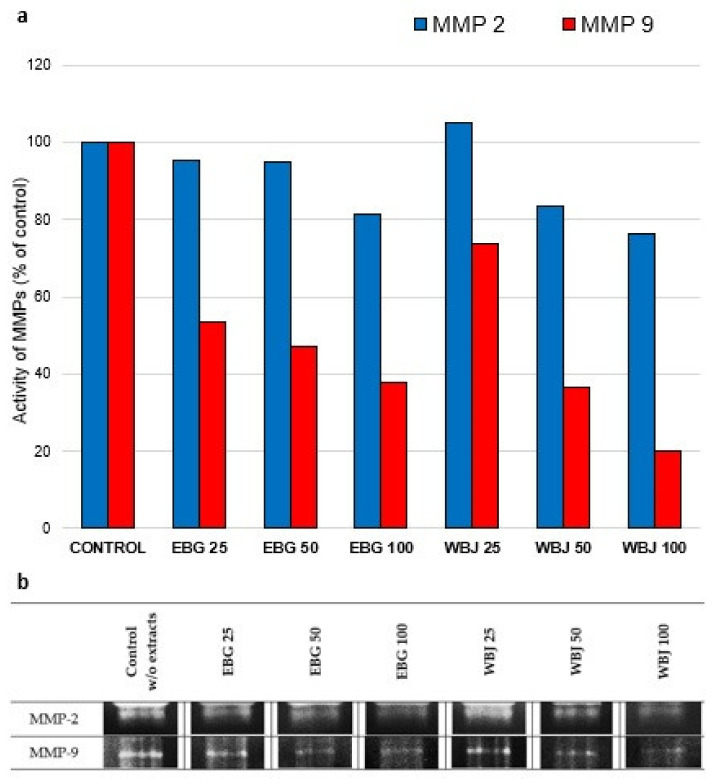
Effect of 70% ethanolic extract of barley grass (EBG) and water extract of young barley juice (WBJ) at doses of 25, 50 and 100 µg/mL on MMP-2 and MMP-9 expression in the culture medium of NHSF line fibroblasts in the “UV 24 h” research model. MMPs secretion was presented as a percentage of control without application of extracts (**a**). Figure (**b**) shows images of gels (medium concentrated by 35-fold; 10 µg of protein).

**Figure 10:**
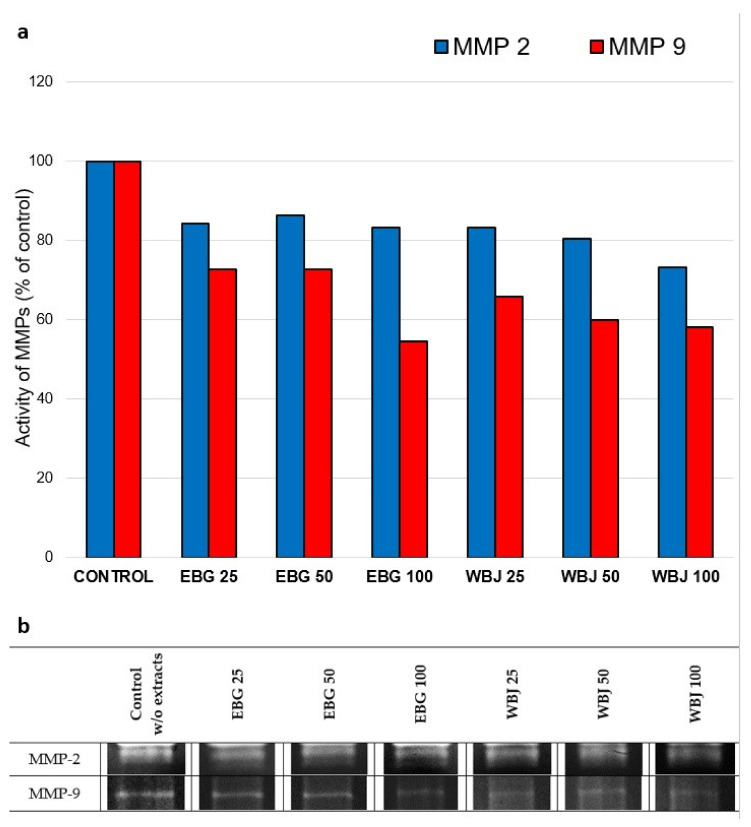
Effect of 70% ethanolic extract of barley grass (EBG) and water extract of young barley juice (WBJ) at doses of 25, 50 and 100 µg/mL on MMP-2 and MMP-9 expression in the culture medium of NHSF line fibroblasts in the “UV 0 h” research model. MMPs secretion was presented as a percentage of control without application of extracts (**a**). Figure (**b**) shows images of gels (medium concentrated by 35-fold; 10 µg of protein).

The results of the effects of 70% ethanolic extract of barley grass (EBG) and water extract of young barley juice (WBJ) on the expression of MMP-2 and MMP-9 secreted by UV-treated human skin fibroblasts obtained by gelatin zymography are summarized in Figure 9 and Figure 10.

Application of EBG extract resulted in the greatest decrease in MMP-2 expression after a dose of 100 µg/mL (by 18.8%) in the model “UV 24 h”. Application of WBJ extract at a dose of 25 µg/mL resulted in a slight (5.1%) increase in MMP-2 activity. The remaining doses of WBJ showed an inhibitory effect against MMP-2, by 16.7% at the 50 µg/mL dose and 23.6% after the 100 µg/mL dose. MMP-9 expression was inhibited after treatment with both extracts at all doses by 46.5–62.0% for EBG and 26.2–80 % for WBJ in a dose-dependent manner (Figure 9a). 

The 100 µg/mL dose of both extracts (EBG and WBJ), in the “UV 0 h” model, had the greatest inhibitory effect against MMP-2 activity, by 16.8% and 26.8%, respectively, relative to the control without extracts. For MMP-9, both types of extracts showed inhibitory effects in a dose-dependent manner. Extract EBG caused a decrease in expression in the range of 27.3–45.5% and extract WBJ 34.3–41.9% (Figure 10a).

### 3.8. The Influence of EBG and WBJ on Cell Migration

**Figure 11:**
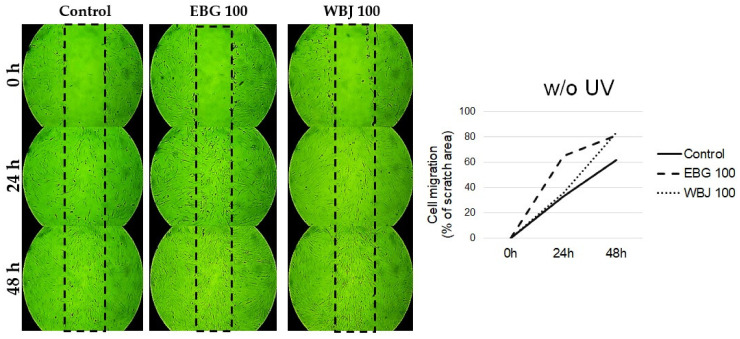
Effect of 70% ethanolic extract of barley grass (EBG) and 100 µg/mL water extract of young barley juice (WBJ) on the migration of NHSF cell line without UV radiation.

**Figure 12:**
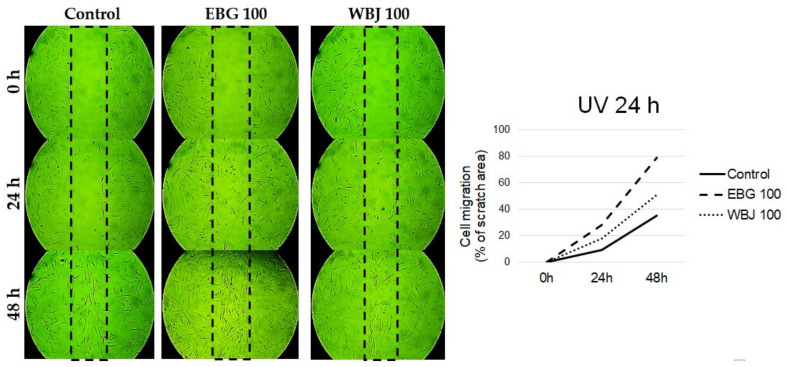
Effect of 70% ethanolic extract of barley grass (EBG) and 100 µg/mL water extract of young barley juice (WBJ) on the migration of NHSF cell line in “UV 24 h” model.

**Figure 13:**
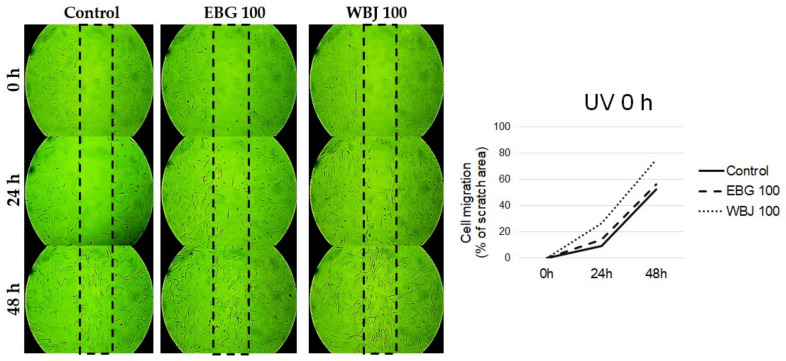
Effect of 70% ethanolic extract of barley grass (EBG) and 100 µg/mL water extract of young barley juice (WBJ) on the migration of NHSF cell line in “UV 24 h” model.

A dose of 100 μg/mL EBG and WBJ was selected to evaluate cell migration using a scratch assay (wound healing assay). The differences in cell migration were compared with the results obtained from control cells without application of extracts and are shown in Figure 11, Figure 12 and Figure 13.

Application of both types of extracts, in the model without UV light application, resulted in an increase in cell migration, both at 48 and 24 h. At 24 h after application of the extracts, the degree of cell migration was higher with the EBG extract, compared to WBJ. At 48 h of the experiment, it was noticed that the cell migration was higher when WBJ extract was applied (Figure 11).

More effective cell migration stimulating effect in the “UV 24 h” model was shown by EBG extract compared to WBJ and control (Figure 12). 

In the “UV 0 h” test model, a stronger effect of WBJ extract on cell migration was observed compared to EBG extract. After 48 h, the number of spaces between cells where WBJ extract was applied was less than that of the control (Figure 13).

### 3.9. Chemical Composition of EBG

A total of 60 components contained in EBG extract were identified in the course of the present study. A list of these constituents is presented in Table 5 and Table 6.

In the first step of the study, the composition of the EBG extract was analyzed, showing the presence of 22 compounds, with carbohydrates dominating (Table 5). Then, the extract was purified from sugar compounds and analyzed again. A total of 38 compounds were identified (Table 6). The main components were 9, 12, 15-octadecatrienoic acid (28.6%), palmitic acid (5.25%), isololiolide (4.35%), α-linolenic acid (3.50%), n-hexadecanoic acid (3.49%).

## 4. Discussion

Human skin is constantly undergoing changes resulting from genetically determined changes in the body overlaid with changes stimulated by extrinsic conditions. The strongest extrinsic factor is UV radiation [31]. Overexposure to sun radiation causes sunburn, degradation of connective tissue, damage to DNA and decrease in immunological response. Chronic exposure to UV interferes with the normal structure and functioning of the skin, which leads to many skin disorders, like photoaging (premature skin aging) and photocarcinogenesis [32]. Exposure to sunlight can also be safe, provided that the skin is optimally protected and the body’s physiological defenses are strengthened, including through a well-balanced diet.

A proper diet has a beneficial effect on the integrity and biological functions of the skin, resulting in the strengthening of its protective role. Many studies have emphasized the special importance of minerals for skin health. Elements such as Zn, Cu and Se play an important role in ensuring proper skin function [33,34]. The young barley products selected for the study, which were commercially available as dietary supplements, did not have information on the label regarding zinc, copper, or selenium content. 

Zn is an essential micronutrient that catalyses enzyme activity (including gelatinases such as MMP-2 and MMP-9), participates in protein synthesis, and regulates gene expression. Moreover, it plays a key role in skin integrity by participating in barrier and immune mechanisms [35,36]. Among the young barley preparations examined in the present study, BJ had the highest micronutrient content. The content of zinc in this preparation was 289.24 ± 12.87 mg/kg, and the recommended daily portion of this product provides 15% of the RVI for zinc [22], which proves that it is a good source of this element. Another important micronutrient showing anti-radical and protective effects on the skin is selenium [37]. Se protects the skin against oxidative stress caused by UV radiation by stimulating the activity of antioxidant enzymes dependent on this element, such as glutathione peroxidase and thioredoxin reductase [33]. This element exhibits anti-aging effects in the skin, influencing the inhibition of wrinkle formation by participating in the repair mechanisms of UV-induced damage [38]. Moreover, due to the anti-inflammatory and antioxidant properties of Se, its role in the prevention of skin cancer, both basal cell carcinoma and squamous cell carcinoma of the skin, has been confirmed [39]. In turn, Cu is characterized by potent antibacterial, antifungal and antiviral activities [40]. It shows a stimulating effect on the proliferation of dermis fibroblasts and participates in the synthesis and stabilization of ECM proteins and angiogenesis. It is a cofactor of SOD, an enzyme involved in protecting the skin from free radical damage, and prevents oxidative damage to cell membranes and lipid peroxidation [41,42]. It is also a cofactor of tyrosinase, the main enzyme involved in melanin synthesis [43]. The content of selenium (52.95 ± 6.71 µg/kg) and copper (14.22 ± 0.34 mg/kg) was evaluated in this preparation. As for copper, its highest content was determined in the chlorophyll preparation (7722.6 ± 6.36 mg/kg) (Table 3). Panizo-Casado et al. (2020) found that in mature barley grain, the average zinc and copper contents were 48.7 mg/kg and 6.74 mg/kg, respectively [44].

In assessing the quality of products for consumption, not only the content of desirable ingredients but also the presence of contaminants is important, which includes toxic elements. The main risks to human health from toxic elements are associated with exposure to lead, cadmium and mercury [45]. 

Exposure to cadmium has an inhibitory effect on the main antioxidant enzymes: glutathione reductase and SOD. This element induces oxidative stress by displacing zinc, copper and iron from antioxidant enzymes, leading to disruption in their metabolism and inhibition of their ROS-neutralizing effects in cells [46,47]. As a carcinogen, cadmium affects cells by disrupting gene expression and DNA methylation errors, blocking apoptosis, and impairing cell differentiation [48]. Cadmium may also act in synergy with other carcinogens such as tobacco smoke and UV radiation [49]. The toxic effects of lead cause significant changes in various biological processes. It directly contributes to ROS production and cellular oxidative-antioxidant imbalance [50,51]. Moreover, mercury impairs intracellular redox homeostasis [52]. 

The assessment of toxic element content carried out in this study allowed us to conclude that the maximum permissible content of cadmium [23], lead [24] and mercury [25] was not exceeded in any of the analyzed products (Table 3). Similarly, in studies covering dietary supplements available on the Polish market from pharmacy and nonpharmacy sales, it was shown that none of the studied preparations exceeded the maximum permissible content of cadmium [53] and lead [54]. However, assessment of toxic element content in dietary supplements from Asia and Europe revealed high cadmium and lead concentrations in one product from Bacopa [55]. As much as 30% of the dietary supplement samples from Lebanon tested showed exceedances of the permissible cadmium content [56].

A number of sunscreen products are available on the cosmetic market, containing organic or inorganic substances that show the ability to disperse and/or absorb UV radiation [57]. In addition to physical and chemical filters, substances of natural origin, which show protective activity against UV radiation, used both externally and in the form of dietary supplementation, are gaining interest [5,58]. 

There are many compounds in foods, especially those of plant origin, that exhibit antioxidant activity. Plant antioxidants, such as carotenoids, polyphenols or vitamins, have the ability to neutralize ROS, which may contribute to stimulate endogenous photoprotection [59]. In addition, phenolic acids, flavonoids, polyphenols or terpenoids may provide protection against radiation penetration into the skin [60,61]. It was shown that some of the polyphenols (taxifolin, silibinin, cyanidin chloride, verbascoside, acacetin, kempferol, quercetin, baicalein, and rutin) had similar protective efficacy against UVB as benzophenone-3. However, several polyphenols (chalcone, resveratrol, leontopodic acid, and trans-ferulic acid) were much more effective UVB blockers [62].

Young barley extract has not yet been studied for its potential photoprotective effect, but there are indications suggesting such an effect of the product. Several studies indicate the antioxidant properties of young barley extracts, which may be mainly due to the antioxidant activity of flavonoids, such as saponarin and lutonarin, present in this product [8]. According to a study conducted by Panizo-Casado et al. (2020), the total polyphenol content of barley grain flour averaged 1.86 mg GAE/g [44], while data on TPC in young barley extracts are limited. The present study showed that water extracts made from the studied products had the highest TPC value, with the highest average polyphenol content of 13.94 ± 0.35 mg GAE/g shown in WBJ extract. Among the 70% ethanolic extracts of young barley products, EBG had the highest TPC value (4.79 ± 0.06 mg GAE/g) (Figure 1). In an experiment conducted by Samsonowicz and Regulska (2018), aqueous extracts of barley seedlings were characterized by TPC values up to 10.7 mg GAE/g and ethanolic extracts up to 10.9 mg GAE/g [63]. A study by Panthi et al. (2020) showed that the methanolic extract of young barley juice had the highest polyphenol content (82.56 mg GAE/g) [64]. 

To quantify the antioxidant capacity of different types of antioxidants present in foods, including dietary supplements and plant material, TAS was evaluated. From this determination, the ability of the test material (e.g., plant extract) to neutralize ROS can be determined [65]. For the extracts made from the tested dietary supplements of young barley, it was shown that EBG showed the highest TAS value among the ethanolic extracts (3.124 ± 0.153 mmol/L), and the water extracts were WBJ (3.353 ± 0.069 mmol/L) (Figure 2). These were higher than the TAS value of ethanolic extract of white quinoa (1.301–1.501 mmol/L) [66].

The analysis of the content of trace elements in the tested products as well as the quality assessment of the tested young barley extracts showed that EBG and WBJ had the most beneficial value of TPC and TAS; therefore, they were selected for further in vitro tests using human skin fibroblast cell line.

In the present study, an attempt was made to evaluate extracts from young barley in terms of protective and restorative effects against UV-treated human skin fibroblast cells. One of the basic requirements for in vitro protective agents is the absence of toxicity to normal cells [67]. Therefore, we first investigated how the extracts alone (without UV application) affect NHSF cell line. We observed a significant increase in fibroblast survival when EBG was applied at 25 µg/mL–by 9.6% (Figure 3a) and WBJ at 100 µg/mL by 15.2% (Figure 3b) compared to the control without extracts. A study by Czerwonka et al. (2017) showed that doses of 2.5 and 5 µg/mL of water extracts from young barley affected fibroblast survival causing an approximately 10% increase in cell survival compared to controls [68]. In addition, our research showed an increase in DNA synthesis in the model without UV light after a dose of 25 µg/mL in comparison to the control for both extracts: EBG (23%; Figure 6a) and WBJ (9%) (Figure 6b). Application of EBG and WBJ at doses of 50 and 100 µg/mL resulted in an increase in the number of cells arrested in S phase, which may explain the effect of EBG extract on the increase in DNA biosynthesis (Figure 7b and Figure 8b). On the other hand, Robles-Escajeda et al. (2013) observed no significant effect of water barley grass extracts on fibroblast proliferation [69]. 

Next, a UV dose of 25 mJ/cm^2^, which is the minimum cytotoxic dose of broadband UVB to fibroblasts, was selected as the optimal dose to evaluate the protective effects of young barley extracts on UV-treated human skin fibroblasts based on the literature information [26]. It should be noted that the minimal erythema dose (MED) value for individuals particularly sensitive to UV radiation (skin phototype I and II according to Fitzpatrick) is 20–25 mJ/cm^2^ [70]. Herman et al. (2014) demonstrated that fibroblasts in vitro do not show significant sensitivity to what are considered environmentally relevant levels of UVB radiation (5 and 10 mJ/cm^2^). After applying these doses, they observed a 10% decrease in cell proliferation compared to unexposed cells [71]. The dose of 25 mJ/cm^2^ UVB radiation selected for this experiment resulted in a 22% reduction in cell survival compared to the non-UV control at 48 h after exposure (Figure 4 and Figure 5). In contrast, other studies have shown that a single exposure of fibroblasts to UVB at a dose of 200 mJ/cm^2^ resulted in a time-dependent decrease in cell survival relative to unirradiated control cells of 19% at 24 h, 24% at 48 h, and 26.2% at 72 h, and 64.5% five days after UVB irradiation [72].

The results of the safety evaluation of the application of young barley extracts to non-UV-treated cells allowed the selection of 70% ethanolic barley grass extract (EBG) and water extract of young barley juice (WBJ) for the evaluation of their photoprotective effects, which were carried out in the “UV 24 h” test model (repair effect) and “UV 0 h” (protective effect). At the same time, chlorophyll extracts (ECh and WCh) were used as a product with photoprotective ability reported in the scientific literature. Cho et al. (2006) found that chlorophyll extract supplementation reduced UV-induced DNA damage and apoptosis in human skin cells [27]. 

EBG extract caused a significant increase in NHSF cell survival in both “UV 24 h” and “UV 0 h” models. All doses used resulted in an increase in cell survival, up to 28.4% at the 50 µg/mL dose (“UV 24 h”) and up to 29.5% at the 25 µg/mL dose (“0 h UV”) (Figure 4a) compared to the corresponding control without extracts. A significant increase in NHSF cells proliferation was also observed with WBJ. The strongest effect of WBJ extract was observed at a dose of 100 µg/mL (by 34%; in the “UV 24 h” model and by 22.2% in the “UV 0 h” model) (Figure 5a). The application of ECh and WCh also resulted in an increase in cell survival, while it was lower than that of the tested young barley extracts (Figure 4b and Figure 5b), indicating that the young barley products were more effective in photoprotection.

One of the main adverse effects of UV radiation on skin is DNA damage, which can be assessed by measuring DNA biosynthesis. Zeng et al. (2014) observed a four-fold decrease in DNA biosynthesis in cells radiated with a dose of 120 mJ/cm^2^, compared to cells without radiation [73]. In our study, application of UVB at a dose of 25 mJ/cm^2^ decreased DNA synthesis in the NHSF cell line by 14% compared to control cells without UV (Figure 6). 

In a model evaluating the protective effect of the extracts (“UV 24 h”), there was a significant increase in DNA biosynthesis after EBG treatment at all doses, ranging from 4% for the 100 µg/mL dose to about 10% for 25 and 50 µg/mL (Figure 6a). WBJ extract also significantly increased DNA synthesis at doses of 25 µg/mL (by 24% relative to the UV 24 h control) and 50 µg/mL (10%) (Figure 6b). The “UV 0 h” model showed a significant increase in DNA biosynthesis after EBG application at doses of 50 µg/mL and 100 µg/mL and was 9% and 13%, respectively (Figure 6a). Application of WBJ extract resulted in a significant decrease in DNA synthesis at all doses and was 9% each for 25 µg/mL and 50 µg/mL and by 7% for 100 µg/mL (Figure 6b).

Changes in UV-treated human skin fibroblasts are also evident in cell-cycle progression. An important response to UVB radiation is an increase in the concentration and phosphorylation of the checkpoint protein p53 in skin fibroblasts, in response to DNA damage, resulting in a transition from temporary to stable cell cycle arrest [74,75]. These modulations prevent radiated cells with high levels of photodimers from transitioning from G1 to S phase [76]. During the present study, we also observed an increase in the number of cells in G1 phase and a decrease in S phase after UV radiation was applied to control cells (without extracts) (Figure 7b and Figure 8b). A similar effect was shown by Straface et al. (2007) using UVB at a dose of 200 mJ/cm^2^; the percentage of cells in G1 phase was significantly increased after UV exposure compared to fibroblasts without radiation treatment [72]. Application of EBG at a dose of 50 µg/mL in the “UV 24 h” research model and EBG (Figure 7b) and WBJ (Figure 8b) at doses of 25 and 50 µg/mL in the “UV 0 h” model resulted in an increase in the number of cells arrested in S phase, which explains the increase in DNA biosynthesis after EBG extract application and indicates its protective and restorative effects. 

In the course of skin photoaging, an increase in the expression of many MMPs, which degrade ECM proteins, is observed. In addition, MMPs play a key role in photocancerogenesis, affecting various processes associated with tumor progression, such as tumor growth, angiogenesis, and metastasis [31,77]. MMP-2 and MMP-9 are responsible for the degradation of type I collagen (MMP-2), elastin and fibronectin [21]. A beneficial effect of the tested extracts was their inhibitory effect on the secretion of MMP-2 and MMP-9. EBG extract showed inhibitory effect on MMP-2 expression in UV-radiated models, with a dose of 100 µg/mL being the most effective and causing a reduction in expression of 18.8% (“UV 24 h”; Figure 9a) and 16.8% (“UV 0 h”; Figure 10a) relative to the control. WBJ extract at a dose of 100 µg/mL also exhibited a reduction in MMP-2 activity, by 23.6% (“UV 24 h”; Figure 9a) and 26.8% (“UV 0 h”; Figure 10a), respectively. MMP-9 expression was inhibited after all doses of both extracts, in a dose-dependent manner. EBG extract inhibited MMP-9 expression by up to 62% in the “UV 24 h” model (Figure 9a) and 45.5% in the “UV 0 h” model (Figure 10a). WBJ extract reduced MMP-9 activity more effectively, compared to EBG, causing an 80% decrease in the “UV 24 h” model (expression 9a) and a 41.9% decrease in the model “UV 0 h” (Figure 10a).

The beneficial effect, both protective and restorative, of the tested extracts was also confirmed by the observed increase in cell migration, which is associated with an increase in cell proliferation. In the present study, the application of young barley extracts resulted in increased migration of fibroblasts not subjected to UV radiation (Figure 11) as well as in both models with UV application (Figure 12 and Figure 13). EBG was more effective in the “UV 24 h” model (Figure 12) while WBJ had a stronger effect in the “UV 0 h” model (Figure 13). 

Evaluation of composition using GC-MS was performed on the most promising young barley extract—EBG. Detailed analysis of the EBG extract showed the presence of many compounds with a wide spectrum of pro-health activity. However, only several of them can influence the skin protective effect. Propanoic acid, 2,3-bis[(trimethylsilyl)oxy]-, trimethylsilyl ester, also called glyceric acid, could induce the proliferation of fibroblasts cells while α-glucosylglyceric acid enhances fibroblast’s synthesis of collagen. Moreover, protective activity on DNA scission exposed on hydroxyl radical and heat-induced protein aggregation have exerted these acids [78]. The research proved that Malic acid improved skin condition in atopic dermatitis through inhibition of MAPK and NF-κB phosphorylation in skin tissue. Additionally, it inhibited the expression and activation of intercellular adhesion molecules, chemokines, and monocyte chemoattractant protein-1 in the thymus. [79]. Malic acid could potentially reduce damage caused by exposition to UV radiation [80]. This combination therapy with aspartic acid induced the necessary paracrine signalling pathway triggering faster regeneration of the damaged tissues of the skin. The activity is based on the replacement of damaged extracellular matrix and enhanced cell proliferation [81]. Aspartic acid significantly improved skin firmness via increased fibrillin-1 and deposition of collagen type IV [82]. This acid could also potentially repair various signs of skin aging [83]. Clinical trials of threonic acid (2,3,4-Trihydroxybutyric acid) sunscreen photoprotective effect performed in human dermis patients showed that threonic acid was a great tool to monitor the oxidative process and control the effectiveness of sunscreen creams. An irradiated group without sunscreen had a lower dermis concentration of acid and a higher erythema index than the other group [84]. The antiphotoaging activity showed derivates of detected quinic acid-3,5-Dicaffeoyl-epi-quinic acid. This effect was tested against skin damage induced by UVA and UVB radiation in human fibroblasts [85,86]. Published studies suggested that inositol may be effective in patients with moderate acne [87]. The next compound detected is 2-methoxy-4-vinylphenol that has anti-inflammatory properties through activation of MAPK, NF-κB suppression and histone H3 acetylation [88]. This property is important because exposure to UV radiation is associated with inflammation and oxidative stress of the skin [89]. Butylated hydroxytoluene detected in extract showed inhibition effect of UV-induction carcinogenesis, erythema, and ornithine decarboxylase after dietary administration [90]. Butylated hydroxytoluene also significantly increased epidermal absorption which suggested photoprotective activity related to a diminution of UV radiation dose [91]. Cinnamic acid was used in cosmetics as a skin-conditioning ingredient and also showed potential as an agent for hyperpigmentation treatment [92]. Moreover, it prevented photoaging induced by UVA radiation via AP-1 inhibition and Nrf2-mediated induction in human fibroblasts [93]. Dodecanoic acid showed potential for use as an anti-inflammatory treatment of acne vulgaris and burn wound colonized by pathogens [94,95]. Moreover, benzaldehyde, 4-hydroxy-3,5-dimethoxy- showed a photoprotective effect and has been used to create a broad-spectrum sunscreen [96]. Neophytadiene inhibited LPS-induced inflammatory response and could be helpful to protect against UV [97]. The study highlighted the role of n-Hexadecanoic acid (palmitic acid) in epidermal morphogenesis and lipid barrier formation [98]. 9,12,15-Octadecatrienoic acid (alpha-linolenic acid) suppressed skin injury induced by UVB radiation and lightened hyperpigmentation induced by ultraviolet of the skin [99]. Additionally, this fatty acid inhibited UV-induced photoaging, tested on hairless mice [100]. 

## 5. Conclusions

The consumption of dietary supplements containing young barley, because of the demonstrated presence of antioxidant minerals (Zn, Cu, and Se), may contribute to enhancing the body’s natural protection against the effects of ROS produced as a result of UV exposure. It should also be emphasized that the tested products were safe in terms of the content of toxic elements, which did not exceed the permitted standards. 

TPC and TAS values also indicate the antioxidant activity of the tested extracts. Due to this property, the use of young barley can support the natural defense mechanisms and also have a positive effect on skin cells, which was confirmed in vitro. 

The use of both 70% ethanolic extract of barley grass (EBG) and water extract of young barley juice (EBJ) is safe because they did not induce cytotoxic effects against human skin fibroblasts in vitro. Moreover, the extracts showed protective (EBG and WBJ) and repairing (EBG) effects against UV-treated cells through beneficial effects on proliferation, stimulation of DNA synthesis, increase in the number of cells arrested in the S-phase of the cell cycle, inhibition of MMP-2 and MMP-9 expression, and acceleration of cell migration.

## Figures and Tables

**Table 1:** Dry mass content [g/mL] in water extracts.

Extract	Sample Volume (mL)	Sample Weight (g)	Water Weight (g)	Water Content (%)	Crude Extract Weight (g)	Crude Extract Content (%)
WBJ	1	1.00	0.92	92.0	0.08	8.0
WBG	1	1.03	0.99	96.1	0.04	3.9
WCh	1	1.00	0.97	97.0	0.03	3.0

Water extract of young barley juice (WBJ), water extract of young barley grass (WBG) and water extract of chlorophyll (WCh).

**Table 2:** Results of quality control of the methods for determination of Zn, Cu, Se, Cd, Pb, Hg in certified reference material.

Element (Unit)	Certified Value ±SD	Certified Value Range	Determined Element Content ± SD (*n* = 6)	Accuracy (%)	Coefficient of Variation (%)
Zn (mg/kg)	34.7 ± 2.7	32.0–37.4	34.8 ± 0.7	0.41	2.1
Cu (mg/kg)	20.4 ± 1.5	18.9–21.9	20.3 ± 0.7	0.54	3.5
Se (µg/kg)	76 *	-	73.1 ± 0.4	3.96	5.5
Cd (µg/kg)	30.2 ± 4.0	26.2–34.2	30.4 ± 0.6	0.49	1.9
Pb (µg/kg)	1780 ± 240	1540–2020	1761 ± 42	1.06	2.4
Hg (µg/kg)	4.9 ± 0.7	4.2–5.6	5.0 ± 0.1	0.81	2.6

* information value. - In the case of an information value, the producer does not specify the range of certified value.

**Table 3:** Mineral content (Zn, Cu and Se) in the studied young barley juice (BJ), barley grass (BG) and chlorophyll (Ch) and percentage of RVI coverage [22].

	Zn		Cu		Se	
Product	Content *x* ± SD (mg/kg)	% RVI (10 mg) *	Content *x* ± SD (mg/kg)	% RVI (1 mg) *	Content *x* ± SD (µg/kg)	% RVI (55 µg) *
BJ	289.24 ± 12.87	15	14.22 ± 0.34	7.1	52.95 ± 6.71	0.48
BG	265.45 ± 17.10	2.6	6.76 ± 0.09	0.7	28.46 ± 3.72	0.05
Ch	232.59 ± 7.07	1.2	7722.6 ± 6.36	386.1	496.45 ± 12.87	0.5

* Regulation (EU) No. 1169/2011.

**Table 4:** Content of toxic elements (Cd, Pb and Hg) in the tested juice of young barley (BJ), barley grass (BG) and chlorophyll (Ch) compared to the maximum limits.

	Cd		Pb		Hg	
Product	Content *x* ± SD (µg/kg)	Norm (µg/kg) *	Content *x* ± SD (µg/kg)	Norm (µg/kg) **	Content *x* ± SD (µg/kg)	Norm (µg/kg) ***
BJ	26.84 ± 2.84	1000	39.58 ± 5.41	3000	9.75 ± 2.19	100
BG	29.49 ± 4.65		0.62 ± 0.08		7.95 ± 1.05	
Ch	52.28 ± 5.33		257.24 ± 18.30		37.67 ± 4.65	

* Regulation (EU) No. 488/2014, ** Regulation (EU) No. 2015/1005, *** Regulation (EU) No. 2018/73.

**Table 5:** Chemical composition of 70% ethanolic extract of young barley grass (EBG).

No.	Components, TMS Derivative	Rt, min	LTPRI Exp	LTPRI Lit	Relative Composition (%)
1	Phosphoric acid	8.42	1284	1285	1.62
2	Butanedioic acid	8.89	1318	1314	0.40
3	Propanoic acid	9.20	1341	1350	0.17
4	2-Piperidinecarboxylic acid	9.62	1372	1378	0.54
5	L-threonine	9.97	1399	1367	0.14
6	DL-Malic acid	11.44	1501	1390	7.73
7	L-Aspartic acid	12.02	1530	1512	0.33
8	Rythronic (Tetronic) acid	12.63	1560	1595	0.13
9	Threonic acid	13.01	1580	1518	0.43
10	Glutamine	14.28	1630	1612	0.10
11	β-D-Galactofuranose	17.89	1731	1852	0.12
12	D-glucofuranose	18.05	1756	1873	0.16
13	2-Keto-l-gluconic acid	19.44	1800	2073	0.38
14	D-Fructose	20.73	1840	1867	1.93
15	d-(-)-Fructose	21.01	1848	1805	8.26
16	L-idofuranuronic acid	22.15	1883	2082	0.29
17	Quinic acid	22.56	1896	1904	0.64
18	Talose	23.59	1929	1970	3.06
19	Mannose	24.87	1968	2052	0.12
20	Myo-Inositol	26.43	2009	2153	0.20
21	Galactopyranose	26.76	2015	2037	3.46
22	Inositol	29.75	2064	2194	0.36

**Table 6:** Chemical composition of 70% ethanolic extract of young barley grass (EBG) after purified from sugar compounds.

No.	Components, TMS Derivative	Rt, min	LTPRI Exp	LTPRI Lit	Relative Composition (%)
1	Levulinic acid	14.69	1175	1130	0.10
2	2,3-dihydrobenzofuran	21.31	1220	1224	0.23
3	1H-Pyrrole-2,5-dione, 3-ethyl-4-methyl (Maleimide)	21.85	1232	1239	0.10
4	Benzoic acid	22.48	1246	1232	0.08
5	Octanoic (Caprylic) acid	23;43	1267	1260	0.12
6	Benzeneacetic (Phenylacetic) acid	24.83	1298	1273	0.16
7	2-Methoxy-4-vinylphenol	25.42	1312	1317	0.52
8	Nonanoic (Pelargonic) acid	27.63	1364	1358	0.05
9	2,5-di-tert-Butyl-1,4-benzoquinone	32.06	1471	1466	0.43
10	Butylated hydroxytoluene	33.71	1512	1503	2.58
11	Dihydroactinidiolide	34.26	1527	1532	0.89
12	Cinnamic acid	34.97	1545	1542	0.31
13	2′,4′-Dimethoxyacetophenone	35.78	1566	1593	0.33
14	Fumaric acid, 2,4-dimethylpent-3-yl ethyl ester	36.03	1572	1528	0.34
15	3-Hydroxy-α-ionene	38.45	1638	1646	0.20
16	Dodecanoic acid (Lauric acid)	39.17	1657	1651	0.10
17	3-Hydroxy-5,6-epoxy-beta-ionone	40.32	1689	1642	0.36
18	9-hydroxy-beta-ionone	40.46	1693	1646	0.22
19	Carbamic acid	40.95	1705	1781	0.12
20	Isololiolide	43.15	1766	1784	4.35
21	Neophytadiene	45.56	1840	1827	1.16
22	Myristic acid	46.05	1855	1850	0.33
23	N, N (82, 81, 95, 43)	46.39	1864	-	0.41
24	N, N (82, 68, 95, 124)	46.98	1883	-	0.32
25	n-Hexadecanoic acid	49.81	1971	1968	3.49
26	Hexadecanal cyclic ethylene acetal	52.21	2048	2030	0.64
27	Palmitic acid	52.48	2057	2039	5.25
28	trans-Phytol	54.42	2122	2104	1.82
29	9,12,15-Octadecatrienoic acid	55.84	2171	2191	28.60
30	9,12,15-Octadecatrienoic acid, ethyl ester,	56.17	2183	2166	0.91
31	Phytol	56.62	2198	2181	1.90
32	α-Linolenic acid	57.82	2241	2218	3.50
33	Stearic acid	58.62	2270	2236	0.35
34	Furan-2-carboxamide, 5-benzoyl-N-(2-dimethylaminoethyl)	65.45	2429	2388	0.02
35	2-Palmitoylglycerol	66.02	2554	2519	0.15
36	Glycerol 1-palmitate	67.44	2511	2482	4.26
37	N, N (44, 43, 55, 73)	69.96	2717	-	0.82
38	N, N (79, 67, 108, 55)	75.42	2962	-	8.75

## Data Availability

The data presented in this study are available in article.
